# Effect of bioaugmentation to enhance phytoremediation for removal of phenanthrene and pyrene from soil with *Sorghum* and *Onobrychis sativa*

**DOI:** 10.1186/2052-336X-12-24

**Published:** 2014-01-09

**Authors:** Mohammad Mehdi Baneshi, Roshanak Rezaei Kalantary, Ahmad Jonidi Jafari, Simin Nasseri, Nemat Jaafarzadeh, Ali Esrafili

**Affiliations:** 1 Department of Environmental Health Engineering, School of Public Health, Iran University of Medical Sciences, Tehran, Iran; 2 Department of Environmental Health Engineering, School of Public Health, Tehran University of Medical Sciences, Tehran, Iran; 3 Center for Water Quality Research, Institute of Environmental Research, Tehran University of Medical Sciences, Tehran, Iran; 4 Environmental Technology Research Center, Ahvaz Jundishapur University of Medical Sciences, Ahvaz, Iran

**Keywords:** Bioaugmentation, *Onobrychis sativa*, Phenanthrene, Phytoremediation, Pyrene, Soil, *Sorghum*

## Abstract

The use of plants to remove Poly-aromatic-hydrocarbons (PAHs) from soil (phytoremediation) is emerging as a cost-effective method. Phytoremediation of contaminated soils can be promoted by the use of adding microorganisms with the potential of pollution biodegradation (bioaugmentation). In the present work, the effect of bacterial consortium was studied on the capability of *Sorghum* and *Onobrychis sativa* for the phytoremediation of soils contaminated with phenanthrene and pyrene. 1.5 kg of the contaminated soil in the ratio of 100 and 300 mg phenanthrene and/or pyrene per kg of dry soil was then transferred into each pot (nine modes). The removal efficiency of natural, phytoremediation and bioaugmentation, separately and combined, were evaluated. The samples were kept under field conditions, and the remaining concentrations of pyrene and phenanthrene were determined after 120 days. The rhizosphere as well as the microbial population of the soil was also determined. Results indicated that both plants were able to significantly remove pyrene and phenanthrene from the contaminated soil samples. Phytoremediation alone had the removal efficiency of about 63% and 74.5% for pyrene and phenanthrene respectively. In the combined mode, the removal efficiency dramatically increased, leading to pyrene and phenanthrene removal efficiencies of 74.1% and 85.02% for *Onobrychis sativa* and 73.84% and 85.2% for *sorghum*, respectively. According to the results from the present work, it can be concluded that *Onobrychis sativa* and *sorghum* are both efficient in removing pyrene and phenanthrene from contamination and bioaugmentation can significantly enhance the phytoremediation of soils contaminated with pyrene and phenanthrene by 22% and 16% respectively.

## Introduction

Polycyclic Aromatic Hydrocarbons (PAHs) are a group of persistent organic pollutants with two or more benzene rings. These compounds are mainly produced via incomplete combustion or pyrolysis of organic compounds [1]. PAHs are hydrophobic compounds that are quickly adsorbed by particulate materials. Therefore, they are abundant in soil [2]. A number of PAHs are known to have mutagenic, carcinogenic, and teratogenic effects [3]. In the recent decades, a broad range of physical, chemical, and biological methods have been applied for the remediation of water and soil contaminated with these hydrophobic organic compounds [4-6].

Phytoremediation and bioaugmentation are among the *in situ* biological methods used for this purpose, which have proved to be cost-effective and environmentally friendly [7]. Phytoremediation is the process of applying plants for removing contaminants from the soil, surface and ground waters, which has been developed during the past decade [8-10]. In this process, organic pollutants, that have been introduced into the soil, can be absorbed by plants and transferred or stored in a non-toxic forms. In addition, increased microbial population, and in turn increased microbial activity, in the rhizosphere can degrade organic pollutants by providing source of nitrogen and carbon through root exudates and sloughing cells [11]. Furthermore, plants modify the soil structure and increase its aeration and humidity. The roots’ exudates contain oxidative enzymes that can contribute to the degradation of PAHs. In addition, plants can physically transfer organic pollutants into their tissues, where they will mineralize them [7].

A number of points should be taken into consideration when using phytoremediation for the removal of PAHs. Firstly, native plants of the target area must be used to ensure that they can tolerate the area’s soil as well as weather conditions. They should also need minimum maintenance, such as the need for fertilizers, to decrease the costs. In addition, they should be able to grow in soils with limited nutrients. Previous research has suggested that legumes are preferable, due mainly to their ability in nitrogen fixation. Finally, the applied plants should have maximum root surface area [12].

The two plant types used in the present study were: 1) one type of Poaceae (i.e. *Sorghum*) and 2) one type of legumes (i.e. *Onobrychis sativa*). The former was selected due primarily to its ability to tolerate severe weather conditions such as drought, extensive root surface, and ability to tolerate pollutants, which the latter was chosen because it’s been native to Iran, especially in central and southern grows abundantly in Rangeland. *Onobrychis sativa* can also prevent soil erosion and grows in nutrient-poor soils which have not been used in phytoremediation. Moreover, its ability in nitrogen fixation and little need for fertilizers and maintenance in general has made more consideration to enhance the phytoremediation efficiency [13,14].

These plants can be easily cultivated in the weather conditions of Iran and can be grown in a wide range of soil and weather conditions. *Sorghum* possesses a fibrous root system and produces an extensive root surface area. *Onobrychis sativa* was also selected because of its ability in nitrogen fixation, which enables it to grow in contaminated soils with high C/N ratios [15].

Microbial degradation is an important process in the dissipation of PAHs in contaminated soils. Bioaugmentation which includes the addition of external microbial species could improve microorganisms' activity to degrade the target toxic compounds. But some studies have reported that bioaugmentation had not significantly effect on bioremediation of low molecular weight PAHs [10,16]. Yu et al. (2005) did not observe any significant difference between natural attenuation and bioaugmentation in phenanthrene biodegradation because of negative interaction between the inoculums and the endogenous microflora [17].

In the present study, a mixture microbial population that adapted with pyrene and phenanthrene was used to investigation of PAHs removal efficiency enhancement in phytoremediation.

## Materials and methods

### Chemical

Phenanthrene and pyrene (purity > 98%) were obtained from Aldrich chemical Co. All other chemicals used in the study were of analytical purity. The seeds used for growth of the two plants were purchased from Pakan Bazar Co., Iran.

### Preparation of pyrene and phenanthrene contaminated soil samples

The soil samples were collected from ranges (0–15 cm depth) of Yasuj city, province of Kohqiluyeh & Buyerahmad, Iran. Sampling procedure was based on the method of Lee et al. (2008) [12]. They were dried, grounded, and screened with a mesh with a pore size of 2 mm. The samples were then spiked with pyrene and phenanthrene at concentrations in the range of 1000–6000 mg/kg of dry soil. To add pyrene and phenanthrene to the soil samples, a known amount of each of the contaminants was dissolved in acetone to evaporate. Afterwards, the pyrene and phenanthrene contaminated soil were mixed uncontaminated soil, so that only 10% of the final samples contain spiked soil, allowing to reach the desired concentrations (Table 1). In order to make sure that the pollutants are evenly distributed in the soil samples, they were well mixed and then screened with a mesh having a pore size of 2 mm. The concentrations of the pollutants were determined in all modes of the experiments, which were not significantly different from the design values. 1.5 kg of the prepared soil was transferred into each pot with a height of 20 cm and a diameter of 15 cm. To ensure complete evaporation of acetone, the pots were kept at laboratory temperature for one week.

**Table 1:** Initial concentration of PAHs in treated soils (mg/kg dry soil)

	**C**_**0**_	**C**_**1**_	**C**_**2**_	**C**_**3**_	**C**_**4**_	**C**_**5**_	**C**_**6**_	**C**_**7**_	**C**_**8**_
Pyrene	0	100	300	0	0	100	300	100	300
Phenanthrene	0	0	0	100	300	100	100	300	300

### Additions of the PAH-degrading bacteria

The bacterial mixture was cultivated on nutrient agars, which were kept at 37°C for 24 hr. The colonies were then dissolved in sterilized deionized water to reach OD_630nm_ = 1 [18]. After cultivating and counting the bacteria, a known amount of the solution was sprayed on the soil samples to reach a bacterial count of 10^6^ per each gram of the soil. Shortly after adding the bacterial, the soil samples were transferred into pots and quickly watered.

### Experimental design

The soil samples were contaminated with pyrene and phenethrene, separately and combined, at different concentrations, as presented in Table 1. For each of the concentration modes presented in this table, six combinations (i.e. natural, with bacteria, *Onobrychis sativa*, *Sorghum*, bacteria + *Sorghum* and bacteria + *Onobrychis sativa*,) were considered (54 treatments), as presented in Table 2. Considering the fact that each of the combinations was repeated three times, a total of 162 pots were used. Experimental design was carried out using the Design Expert 7 in the full factorial mode.

**Table 2:** Treatment of experimental design

	**T**_**0**_	**T**_**1**_	**T**_**2**_	**T**_**3**_	**T**_**4**_	**T**_**5**_
Natural attenuation	+					
Bioaugmentation		+			+	+
*Sorghum*			+		+	
*Onobrychis S.*				+		+

In each of the 162 pots, 1.5 kg of the prepared soil from the 54 treatments was poured. In addition, six seeds of each plant with the same density were cultivated in a depth of 15 cm. The plants were watered every other day with deionized water in 60% of the water holding capacity. This was done for one week in the laboratory until germination took place.

Then, three similar plants were kept in each pot and the rest of them were removed. The pots were then transferred to a place with conditions similar to those in the field. The pots were randomly moved every third day.

### Determination of phenanthrene and pyrene in soil

For extraction of PAHs from soil, method of 3550B EPA with some modification has been described by PAN et al. (2008) was used [1]. The soil samples in the pots were carefully collected, homogenized, and passed through a mesh having a pore size of 2 mm. two grams of the soil was transferred to a centrifuge tube and mixed with 20 g dehydrated Na_2_SO_4_. 10 mL dichloromethane was then added to it and kept at 40°C in a bath ultrasonic for 1 hour. Afterwards, the tubes were centrifuged at 4000 rpm, and 3 mL of the supernatant was passed through silica gel column. It was then diluted with a 1:1 Hexane and dichloromethane solution. The diluted samples were evaporated at 40°C and reached to the final volume of 2 mL with methanol. The samples were then passed through micro filters with a pore size of 0.22 μm. The chemical analysis was then conducted using a high performance liquid chromatography (HPLC, CECIL 4100, USA) instrument having a C_18_ column with a length of 25 cm, an internal diameter of 4.2 mm, a UV/VIS is detector, and a mobile phase of methanol: water v/v 80:20), operating at a flow rate of 1 mL/min at a wavelength of 220 nm.

### Microbial count

Plant less soil samples were used for bacteria count. In case the soil samples contained plants, the rhizospheric soil attached to the roots was used. The samples were kept at 4°C until the time of bacterial count, which was performed using the dilution series method. First, 1 g of the soil sample was mixed with 100 mL sterilized deionized water to reach a dilution of 10^-2^. Then, 1 mL of this suspension was added to 9 mL of sterilized deionized water to obtain dilution series up to 10^-6^. 1 mL of the last three dilutions was cultivated on nutrient agar using the pour plate method. The plates were kept for 24 hr and then the number of colonies was counted and reported as the number of colony-forming units per g dry soil (CFU/g) [19].

### Statistical analysis

Analysis of variance with post hoc Duncan’s multiple test and correlation using SPSS Version 16.0 software package.

## Results and discussion

**Figure 1:**
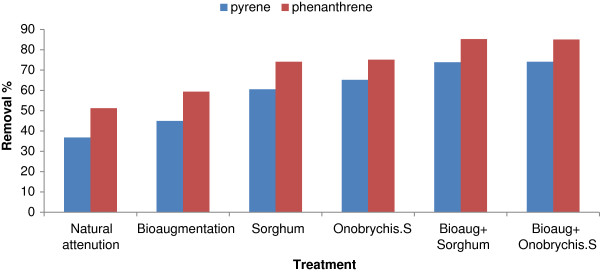
Percentage of degradation for PAHs in different treatment.

Combination of bioaugmentation and phytoremediation in six treatments were considered for investigation of PAHs biodegradation in nine contaminated levels of soil. Figure 1 illustrates the average removal efficiency for pyrene and phenanthrene in all of the treatment. Although the collected soil samples did not have prior contamination, a rather significant level of degradation was achieved in the natural treatment for both pyrene and phenanthrene. This high level of degradation is probably due to the increased microbial population of the soil caused by provision of appropriate level of humidity by watering. Because soil humidity could increase the microbial activity of soil by increasing the population and activity of soil microflora and hence degradation of pollutants has been heightened [20].

However, the level of degradation was dependent on the pollutant, with phenanthrene being degraded 1.4-fold as high as pyrene. This finding was consistent with those of the previously conducted studies, suggesting that PAHs with high molecular weight have higher resistance against degradation than PAHs with low molecular weight. The higher level of dissipation achieved for phenanthrene is due mainly to its higher volatility and biodegradability, compared to pyrene [21]. Zhang et al. (2010) reported that the degradation of phenanthrene in natural condition was significantly more than that for pyrene [2]. Bioaugmentation enhanced the removal efficiency for both pyrene and phenanthrene (8.1% for pyrene versus 8.2% for phenanthrene) (Figure 1). Results from previous studies also suggested increased removal of pollutants from the soil due to bioaugmentation [22,23]. Teng et al. (2010) reported that bioaugmentation enhanced the PAHs removal efficiency [24]. Mohan et al. (2008) also observed a 24% increase in the removal efficiency of pyrene from the soil due to bioaugmentation [25].

The more removal efficiency in bioaugmentation could be occurred due to lack of microorganisms able to degrade pollutants. So, bioaugmentation by bacteria with this ability may improve the degradation of both pollutants. Differences between the increase in removal efficiency of pyrene and phenanthrene may be occurred due to the difference in biodegradability of these pollutants. Pyrene has lower biodegradability and higher toxicity which affects soil microflora and therefore its biodegradation has been reduced in natural condition. However, more biodegradation of pyrene has been observed by application of adapted bacteria which is caused more biodegradation efficiency of the pollutant [25].

Phytoremediation also had a significant effect on the removal efficiency of pyrene and phenanthrene (P < 0.05). The removal efficiencies of pyrene and phenanthrene were 60.4% and 74.1% when using *Sorghum* (23.6% and 22.9% higher than those of the natural group) and 65.2% and 75.1% when using *Onobrychis sativa* (28.4% and 23.9% higher than those of the natural group). *Onobrychis sativa* had a better performance than *Sorghum* in removing pyrene (P < 0.05), while there was no statistically significant difference in their removal efficiencies for phenanthrene. This suggests that *Onobrychis sativa* can perform more efficiently than *Sorghum* in removing pyrene from the soil. It was also observed that phytoremediation is more effective for pyrene than for phenanthrene.

Results from previous research applying a wide range of plants for the phytoremediation process verify its high performance in removing contaminants from the soil [26-28]. The study by Liste and Alexander (2000) carried out on nine different plant types revealed that phytoremediation can increase the removal efficiency of phenanthrene from 40% (without applying any plants) to 74% (with plants) [29]. In another study conducted by Lu et al. (2010) the removal efficiency of pyrene increased 28% increase due to the phytoremediation process [30]. Lee et al. (2008) by applying four types of plants of legumes and grasses families has removed phenanthrene and pyrene from the soil by 99% and 77-94%, respectively. This study suggested that legumes had a higher performance than grasses in removing pyrene, while no difference was observed in their performance for the removal of phenanthrene. This finding is consistent with the results from the present study [12]. The difference between results of studies could be related to the type of plant, soil and weather condition. In some studies which high degradation has been reported, experiments were conducted in a controlled condition and a greenhouse which the condition for plant growth was better. However, this study was applied in the field condition which could affect the plant growth. The effect of applying plants on the degradation of contaminants may stem from the effects of plants’ roots on the condition of surrounding soil. This effect may include increased the microbial activity of rhizosphere and the herbal enzymes secreted in the root area, which can act as degradation intermediacies [12].

Since PAHs are hydrophobic compounds, their release from the soil matrix to aqueous phase happens fairly slowly, which leads to the fact that they remain in the soil for long periods of time. Therefore, application of a single process for removing them from the soil might be quite difficult and time-consuming [2]. In the present work, likewise, only 36.8-65.7% of pyrene and 51.8-75.1% of phenanthrene were removed from the soil by applying a variety of processes for 120 days. This suggests that although these processes have had a significant impact on the removal of pyrene and phenanthrene, a significant level of these pollutants have still remained in the soil therefore, a combination of the above process was used to enhance their removal efficiency. Using the multi-technique phytoremediation process, higher removal efficiencies were observed for pyrene and phenanthrene. When bioaugmentation was combined with phytoremediation with *Sorghum*, the mean removal efficiencies of phenanthrene and pyrene increased up to 85.2% and 73.8%, respectively, compared to removal efficiencies of 74.1% and 60.4% when *Sorghum* was applied alone (11.1% and 13.4% increase).

Similar results were also observed when combining bioaugmentation with *Onobrychis sativa*; in this treatment, the removal efficiencies were 85% and 74.1% for phenanthrene and pyrene, respectively. For both plants, the combined mode with bioaugmentation resulted in significantly higher removal efficiencies than those of the application of each plant alone (Figure 1), which is consistent with the results from the previous works [31]. The difference between removal efficiency of phytoremediation and bioaugmentation is related to the type of plant and experiments condition (greenhouse or field). The removal efficiency of bioaugmentation may be improved by addition of microorganism which is not found in soil. Interaction effect between the plant and bioaugmentation on the pyrene removal was significant (P < 0.05), however for phenanthrene was not (Table 3). Yu et al. (2010) found that the removal efficiency of phenanthrene from the soil in the combined mode with bioaugmentation was 9% more than applying phytoremediation alone. Also, similar result (6% increases) was observed for pyrene [7]. However, Xu et al. (2010) did not observe any improvements in the removal efficiency of pyrene and phenanthrene when applying a combination of phytoremediation and bioaugmentation [32].

**Table 3:** ANOVA for PAHs removal efficiency

**Factor**	**df**	**Mean square**	**F-value**	**P-value**
**Pyrene**				
A:Bioaugmentation	1	5970.31	950.37	<0.0001
B:Plant	2	27392.16	4360.2	<0.0001
AB	2	127.28	20.26	<0.0001
**Phenanthrene**				
A:Bioaugmentation	1	4536.58	584.75	<0.0001
B:Plant	2	17286.48	2228.17	<0.0001
AB	2	10.39	1.34	0.2647

The enhanced removal efficiency might be attributed to the heightened microbial activity caused by the provision of favorable conditions in the rhizosphere of the plant. Plants increase nutrient availability by secreting cationic chelators, organic acids, or specific enzymes and may also increase the bioavailability of the contaminant. This suggests that root exudates increase degradation of contaminant by increasing contaminant availability and (or) stimulating microbial activity. Plants also secrete surfactants (i.e., lipids and sterols), which lubricate the root as it passes through soil. Surfactants reduce surface tension and solubilize contaminants, thereby increasing contaminant bioavailability [33]. It could be concluded that by plant growing, population of added microorganisms in rhizosphere area were getting more. On the other hand, root exudates affect genetic structure of microbial population and increase diversity of the microbial population. This diversity even in the absence of population growth, improve the degradation of pollutants [2].

**Figure 2:**
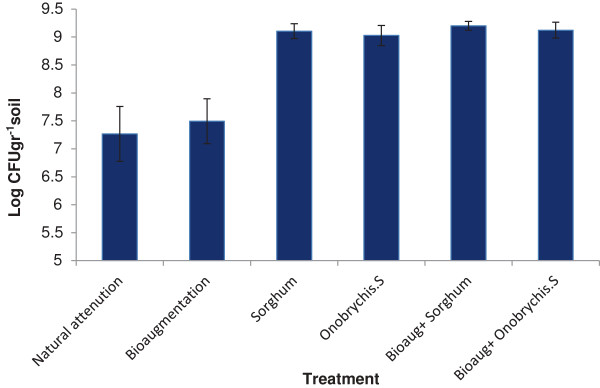
Bacterial population in different treatment.

The bacterial count in different treatment (i.e. natural and bioaugmentation) was in the range of 10^7^ CFU/g of the dry soil, while being in the range of 10^9^ CFU/g of the dry soil in the rhizosphere of the plants (Figure 2). This increases bacterial population was due to the reproduction of bacteria in the presence of plant roots, because plants roots exudates a combination of amino acids, organic acids, enzymes, carbohydrates, and sugars, which can be used as sources of carbon and energy by the bacteria present in the rhizosphere [34]. The results of statistical tests indicated that the presence of plants significantly increased the bacterial population (P < 0.01), while bacterial population did not significantly differ in different treatment where plants had been used.

**Figure 3:**
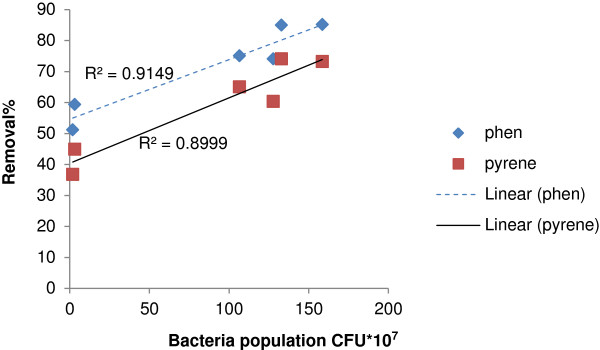
Correlations between percentage of PAHs degradation and bacterial population.

In the absence of plants, bioaugmentation also significantly increased the bacterial population (P < 0.005), while its effect was not significant in the presence of plants. The relationship between Percentage of PAHs Degradation and Bacterial population shows a positive significant Correlations (r = 0.956 and 0.948 for phenanthrene and pyrene respectively) (Figure 3). The results from the previously conducted studies are in agreement with the findings of the present work [17,35]. In the study carried out by Janbandhu et al. (2011) the bacterial population in the rhizosphere was 5–7.5 times larger than in the non-rhizosphere soils [36]. In the study of Gao et al. (2006) an increase in the initial concentration of phenanthrene and pyrene increased the bacterial population in the rhizosphere, whereas decreasing that of the soil [37]. In another study, increasing the initial concentrations of phenanthrene and pyrene increased the bacterial population present in the rhizosphere as well as those in soils with or without plants [30].

## Conclusions

The results from the present work suggested that the application of *Onobrychis sativa* and *Sorghum* can enhance the removal of phenanthrene and pyrene from the soil. Applying multi-technique phytoremediation system can also enhance the removal efficiency of the contaminants, i.e. phenanthrene and pyrene. The use of bacteria can increase the efficiency of phytoremediation for the removal of phenanthrene and pyrene. The favorable effect of bioaugmentation is due to its dissolving and desorption capabilities, which increases the bioavailability of contaminants present in the soil. The regression analysis indicated that the removal efficiency of phenanthrene and pyrene have a significant association with the bacterial population of the soil. Finally, it is advisable to conduct further research to evaluate the efficiency of other native plants in removing PAHs from the soil as well as to provide more insight into the mechanism of PAHs degradation and to determine the amount of PAHs intake by the plants.

## Competing interests

The authors declare that they have no competing interests.

## Authors’ contributions

The overall implementation of this study including design, experiments and data analysis, and manuscript preparation were the results of efforts by corresponding author. All authors have made extensive contribution into the review and finalization of this manuscript. All authors read and approved the final manuscript.
